# Wear Simulation of Ceramic-on-Crosslinked Polyethylene Hip Prostheses: A New Non-Oxide Silicon Nitride versus the Gold Standard Composite Oxide Ceramic Femoral Heads

**DOI:** 10.3390/ma13132917

**Published:** 2020-06-29

**Authors:** Makiko Yorifuji, Saverio Affatato, Toshiyuki Tateiwa, Yasuhito Takahashi, Takaaki Shishido, Elia Marin, Matteo Zanocco, Wenliang Zhu, Giuseppe Pezzotti, Kengo Yamamoto

**Affiliations:** 1 Department of Orthopedic Surgery, Tokyo Medical University, 6-7-1 Nishi-Shinjuku, Shinjuku-ku, Tokyo 160-0023, Japan; yorifujiko@gmail.com (M.Y.); tateiwa@tokyo-med.ac.jp (T.T.); yasuhito@tokyo-med.ac.jp (Y.T.); takaakishishido@aol.com (T.S.); pezzotti@kit.ac.jp (G.P.); 2 Laboratorio di Tecnologia Medica, IRCCS Istituto Ortopedico Rizzoli, Via di Barbiano 1/10, 40136 Bologna, Italy; 3 Ceramic Physics Laboratory, Kyoto Institute of Technology, Sakyo-ku, Matsugasaki, Kyoto 606-8585, Japan; elia-marin@kit.ac.jp (E.M.); matteo.zanocco@gmail.com (M.Z.); wlzhu@kit.ac.jp (W.Z.)

**Keywords:** hip simulator, wear, Raman microspectroscopy, vitamin-E-diffused crosslinked polyethylene, BIOLOX^®^delta, Silicon Nitride.

## Abstract

The purpose of the present study was to compare the wear behavior of ceramic-on-vitamin-E-diffused crosslinked polyethylene (Vit-E XLPE) hip bearings employing the gold standard oxide ceramic, zirconia (ZrO_2_)-toughened alumina (Al_2_O_3_) (ZTA, BIOLOX^®^*delta*) and a new non-oxide ceramic, silicon nitride (Si_3_N_4_, *MC^2^*^®^). In vitro wear test was performed using a 12-station hip joint simulator. The test was carried out by applying the kinematic inputs and outputs as recommended by ISO 14242-1:2012. Vitamin-E-diffused crosslinked polyethylene (Vit-E XLPE) acetabular liners (E1^®^) were coupled with Ø28-mm ZTA and Si_3_N_4_ femoral heads. XLPE liner weight loss over 5 million cycles (Mc) of testing was compared between the two different bearing couples. Surface topography, phase contents, and residual stresses were analyzed by contact profilometer and Raman microspectroscopy. Vit-E XLPE liners coupled with Si_3_N_4_ heads produced slightly lower wear rates than identical liners with ZTA heads. The mean wear rates (corrected for fluid absorption) of liners coupled with ZTA and Si_3_N_4_ heads were 0.53 ± 0.24 and 0.49 ± 0.23 mg/Mc after 5 Mc of simulated gait, respectively. However, after wear testing, the ZTA heads retained a smoother topography and showed fewer surface stresses than the Si_3_N_4_ ones. Note that no statistically significant differences were found in the above comparisons. This study suggests that the tribochemically formed soft silica layer on the Si_3_N_4_ heads may have reduced friction and slightly lowered the wear of the Vit-E XLPE liners. Considering also that the toughness of Si_3_N_4_ is superior to ZTA, the present wear data represent positive news in the future development of long-lasting hip components.

## 1. Introduction

Ultra-high molecular weight polyethylene (UHMWPE, henceforth simply PE) is one of the most promising and widely used bearing materials in total hip arthroplasty (THA). Nevertheless, articulation wear of PE components results in osteolysis and eventual implant loosening. This has historically been the principal problem limiting the longevity of THA [1]. Significant efforts have been made to improve PE wear resistance without compromising fatigue strength, toughness, and oxidative stability [2,3]. One recent approach has been the incorporation of synthetic antioxidant vitamin E (α-tocopherol, Vit-E) into highly crosslinked UHMWPE (XLPE) by blending it into PE powder before consolidation [4] or by diffusing it into already consolidated and crosslinked forms of PE [5].

Another method of minimizing PE wear has been the use of ceramic femoral heads. The selection of aluminum oxide (alumina; Al_2_O_3_) ceramics as a biomaterial was based on its unsurpassed biological safety and stability in the human body, its hardness, and, more importantly, its superior surface smoothness that ensures minimal damage to the “soft” PE liner [6,7,8]. Zirconia (ZrO_2_)-toughened Al_2_O_3_ (ZTA), a matrix composite, known by the trade name of BIOLOX^®^*delta* (CeramTec AG, Plochingen, Germany) was developed for joint applications in 2003. With more than 3.8 million implanted ZTA femoral heads, BIOLOX^®^*delta* is currently recognized as the gold standard ceramic bearing in THA. With over 10 years of follow-up, it has exhibited excellent clinical results [9]. However, a potential drawback of ZTA is its environmentally induced structural destabilization (i.e., tetragonal-to-monoclinic phase transformation in ZrO_2_), leading to surface roughening as well as microcracking [10,11]. Thus, the necessity of elongating the longevity of artificial joints beyond the lifetime of patients is driving the need for a continuous evolution in materials—from conventional to more sophisticated ceramics. In this context, a non-oxide bioceramic, silicon nitride (Si_3_N_4_), was developed as an innovative bearing material in 2008. Although Si_3_N_4_ has a favorable combination of properties, such as high strength, biocompatibility, and fracture toughness [12,13], its wear performance as an arthroplastic bearing has yet to be fully evaluated. 

The purpose of the present study is to compare the wear behavior of ceramic-on-Vit-E XLPE hip bearings by employing the gold standard ZTA oxide ceramic (i.e., BIOLOX^®^*delta*) and a new non-oxide Si_3_N_4_ ceramic in a standard 12-station hip joint simulator. Although the tribological behavior of BIOLOX^®^*delta* has already been the object of a number of detailed studies as tested vs. a variety of different sliding counterparts [14,15,16,17,18,19,20,21,22], this is the first reported direct comparison of the wear performance of Vit-E XLPE liners against ZTA and Si_3_N_4_ femoral heads.

## 2. Materials and Methods

### 2.1. Specimens Tested

The tested Vit-E XLPE acetabular components were E1^®^ Ringloc Max-Rom liner produced by Zimmer Biomet G.K. (Tokyo, Japan), which had a thickness of 8.9 mm, head size of Ø28 mm, liner size 24, and cup size of Ø(54/56) mm. Manufacturing of these components started with GUR1050 resin (Celanese, Inc., Florence, KY, USA). Liquid Vit-E was infused into isostatically compression-molded and crosslinked forms of the PE and the components were subsequently annealed (24 h at 130 °C) to homogeneously diffuse it into the PE structure. Intermolecular crosslinking was achieved by 100-kGy gamma irradiation and final sterilization was performed by gamma irradiation in an inert atmosphere (25–40 kGy dose in argon). 

**Figure 1:**
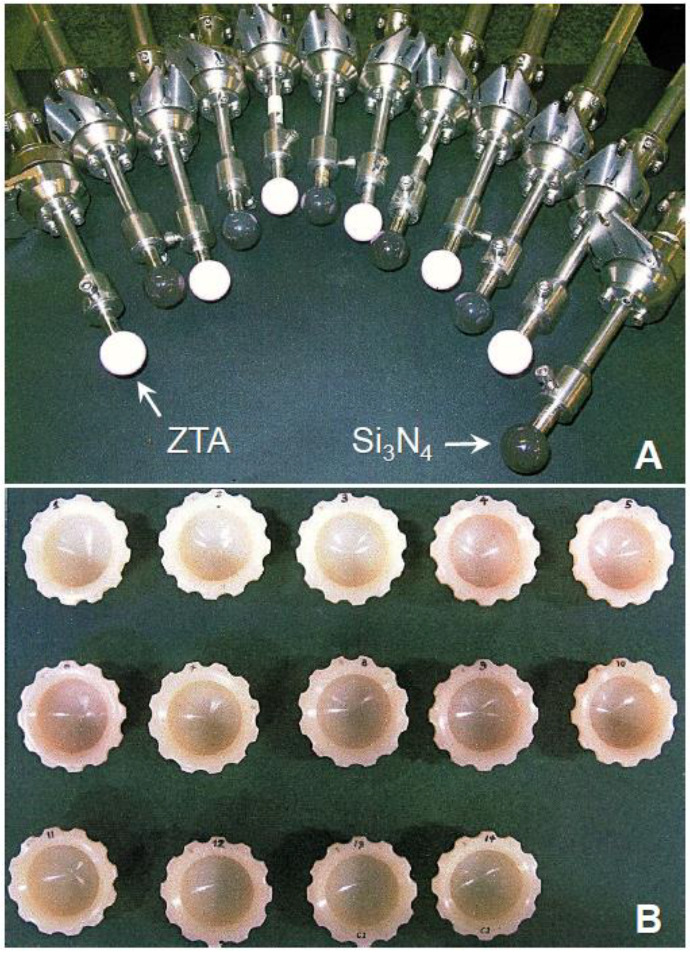
Photographs of (**A**) the tested femoral heads (Ø28 mm), zirconia (ZrO_2_)-toughened alumina (Al_2_O_3_) (ZTA) (BIOLOX^®^*delta*) and Si_3_N_4_ (*MC^2^*^®^), and (**B**) vitamin-E-diffused crosslinked polyethylene (Vit-E XLPE) cups.

The ceramic femoral heads were Ø28-mm ZTA (BIOLOX^®^*delta*; CeramTec AG, Plochingen, Germany) and Si_3_N_4_ (*MC^2^*^®^; Amedica, now SINTX Technologies, Inc., Salt Lake City, UT, USA) (Figure 1). The former oxide ceramic consists of an 82 vol.% Al_2_O_3_ reinforced by 17 wt.% yttria (Y_2_O_3_; 1.3 mol.%)-stabilized ZrO_2_ (Y-TZP), 0.5 wt.% chromium oxide (Cr_2_O_3_) and 0.5 wt.% strontium oxide (SrO) [9]. The mean sizes of the Al_2_O_3_ and ZrO_2_ grains were about 1.5 and 0.5 μm, respectively. The latter non-oxide ceramic consists of about 90 vol.% Si_3_N_4_ sintered with 6 wt.% Y_2_O_3_ and 4 wt.% Al_2_O_3_ [12,13]. The fabricated Si_3_N_4_ had a uniform microstructure of fine and elongated grains with a mean grain width of about 1.5 µm.

### 2.2. Experimental Wear Testing Details

Vit-E XLPE acetabular liners (*n* = 12) were coupled with the two different types of ceramic femoral heads (ZTA and Si_3_N_4_; *n* = 6 each). All acetabular liners were pre-soaked prior to wear testing and were weighed every four days until the weight change between the last two measurements exhibited less than 1% change as per ISO 14242.

Wear testing was performed using a 12-station hip joint simulator (IORSynthe, Bologna, Italy) [2]. The test was carried out by applying the kinematic inputs and outputs as recommended by ISO 14242-1:2012. The simulator utilized hydraulic actuators to apply the cyclic vertical compressive loads (oscillating between 300 and 3000 N perpendicular to the acetabular components as recommended by the standard). The lubricant was 25 vol.% newborn calf serum balanced with distilled water, with 0.2 wt.% sodium azide to retard bacterial growth and 20 mM ethylene-diamine-tetracetic acid (EDTA) to minimize precipitation of calcium phosphate. The weight loss of each acetabular liner was determined every 0.4 million cycles using a semi-microbalance (Sartorius Cubis Mse 225 S-000-DU, Goettingen, Germany) with a sensitivity of 0.01 mg and an uncertainty of 0.01 mg. During the length of the test, which was set up to 5 million cycles (Mc), the samples were weighed at regular intervals and the wear trends determined from the weight loss of each liner, corrected by an acetabular soak control. The wear rates, calculated from the steady-state slopes of the function linking weight loss of the acetabular cups to the number of cycles, were obtained using least squares linear regression.

### 2.3. Surface Roughness Measurements

The surface roughness of all 12 ceramic femoral heads was measured using a contact profilometer (Hommel Tester T8000, Hommel Werke, Koeln, Germany) equipped with a diamond stylus (tip radius 0.020 mm). Scanning operations were performed at ten points for each femoral head—one point on the pole and three random points on three different planes identified according to a standardized protocol [23]. Sampling lengths were taken using a cut-off of 0.08 mm and tracing length of 0.48 mm. Three main indicators were used to characterize the roughness of the femoral heads, namely *R*_a_, *R_t_*, and *R*_sk_. *R*_a_ represents the arithmetical mean height of the roughness irregularities (i.e., peaks and valleys) from the mean line. *R_t_* is defined as the height difference between the highest peak and lowest valley in the tracing length for the entire analyzed area. *R*_sk_ is the skewness and represents the degree of symmetry of the surface heights about the mean plane; the best surface has *R*_sk_ equal to 0. 

### 2.4. Surface Phase Contents and Stress Measurements

Surface crystallinity percentages (*α_c_*) of the Vit-E XLPE liners and transformed percentages (i.e., monoclinic volume percentages in ZrO_2_; *V_m_*) of ZTA heads were characterized by Raman microspectroscopy before and after the 5 Mc wear simulation. Surface residual stresses (σ) were also assessed in the Al_2_O_3_ matrix phase of the ZTA and on the Si_3_N_4_ after completion of the wear simulation.

Raman characterizations employed a triple monochromator (T-64000, Jobin-Ivon/Horiba Group, Kyoto, Japan) equipped with a charge-coupled device (CCD) detector. The excitation source was a 488 nm Ar-ion laser (GLG3103, Showa Optronics Co., Ltd., Tokyo, Japan) yielding a power of 30 mW. The in-plane and in-depth spatial resolution of the Raman probe was confined to approximately 1 and 6 µm, respectively, by means of a 100× objective lens with a confocal pinhole (Ø100 µm) placed in the optical circuit. An automated sample stage was employed to collect square spectral maps (each map size was 50 × 50 µm^2^ with a square mesh of 5 µm steps) in each as-received (unworn) and worn surface. 

A mixed Gaussian–Lorentzian curve fit was applied for the deconvolution of the recorded spectra into sub-bands, and subsequently the integrated intensities and peak positions were calculated from the deconvoluted sub-bands. The computational details for *α_c_*, *V_m_*, and σ are described in previous publications [24,25,26,27].

### 2.5. Statistical Analysis

A non-parametric Mann–Whitney U test was performed with the aid of OriginPro 2016 software (OriginLab Corporation, Northampton, MA, USA). It was used to test for statistically significant differences in the analyzed data (weight loss, *R*_a_, *R_t_*, *R*_sk,_ *α_c_*, *V_m_*, σ) both between specimens and before and after wear testing. The statistical differences in the above comparisons were considered significant at the *p* < 0.05 level.

## 3. Results

**Figure 2:**
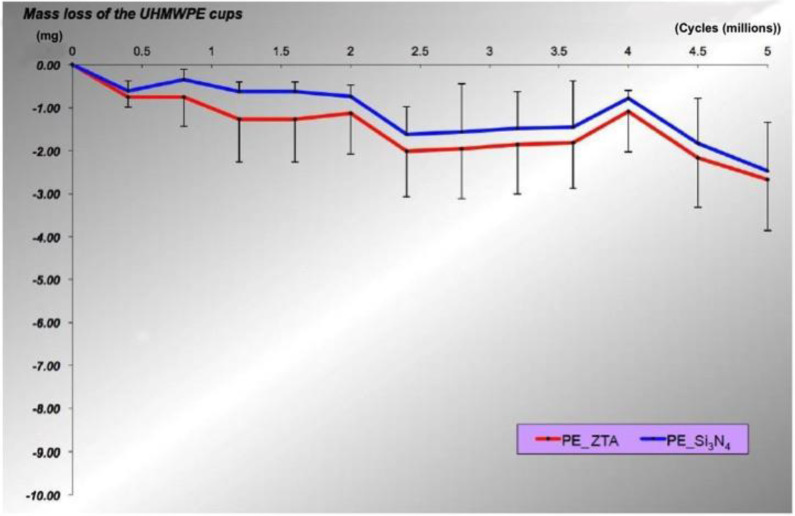
Comparison of total mass loss over 5 million cycles (Mc) of simulated gait between PE_ZTA and PE_ Si_3_N_4_ bearing couples.

All the acetabular liners and femoral heads completed the planned 5 Mc test. The weight of Vit-E XLPE liners was found to decrease along the series ZTA > Si_3_N_4_ throughout the wear test (Figure 2). 

The mean wear rates corrected for fluid absorption of the Ø28-mm Vit-E XLPE liners coupled with ZTA and Si_3_N_4_ heads were 0.53 ± 0.24 and 0.49 ± 0.23 mg/Mc, respectively, after completion of 5 Mc of simulated gait. However, no statistically significant differences were found between the different head groups (*p* = 0.818) (Table 1).

**Figure 3:**
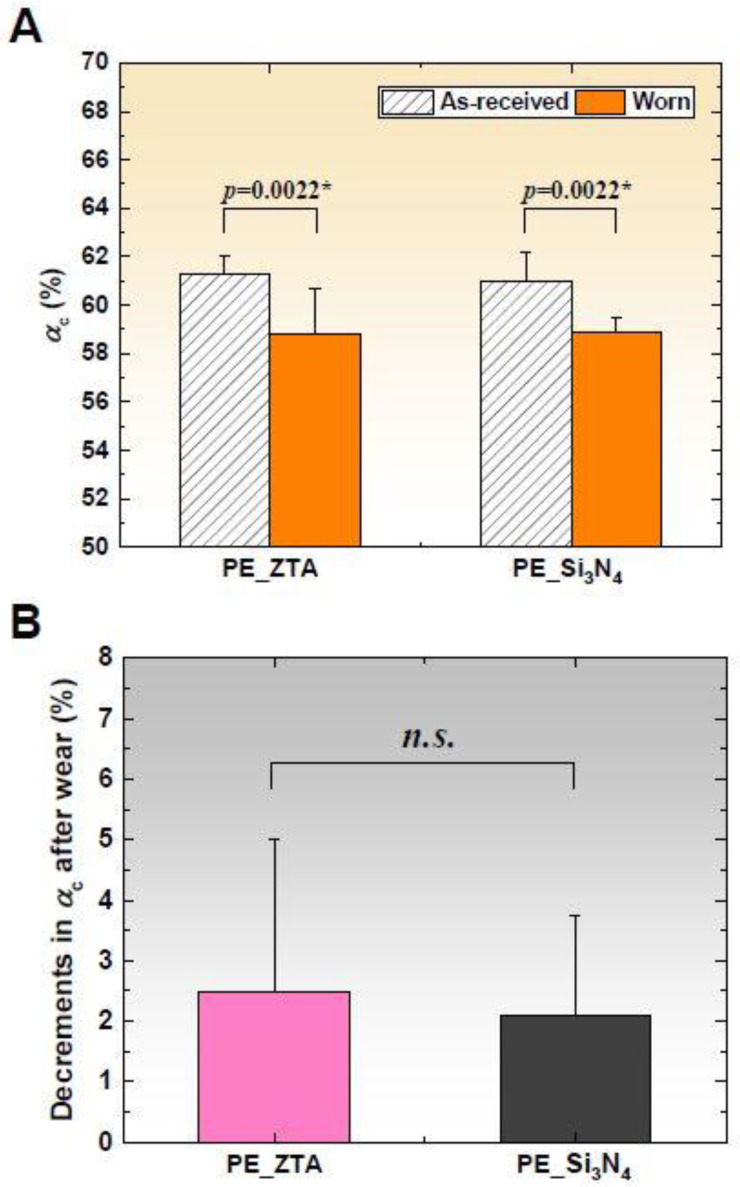
(**A**) Comparison of surface crystallinity (*α*_c_) of Vit-E XLPE acetabular liners coupled with ZTA and Si_3_N_4_ before and after 5 Mc wear simulation; and (**B**) comparison of decrements in *α*_c_ after wear. The asterisks and *n.s.* (not statistically significant) represent *p* < 0.05 and *p* > 0.05, respectively.

Figure 3A compares the surface crystallinity (*α_c_*) for the as-received and worn surfaces of the Vit-E XLPE liners. The mean *α_c_* values significantly decreased after wear testing in both bearing couples (*p* = 0.0022 and 0.0022 in ZTA-on-XLPE and Si_3_N_4_-on-XLPE). However, no statistically significant differences were found in *α_c_* between both pairings (*p* = 0.8983) (Figure 3B).

**Figure 4:**
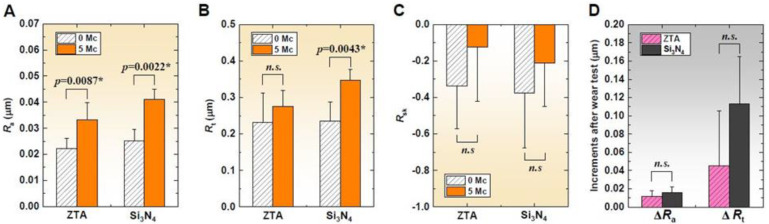
Comparison of surface roughness of ZTA and Si_3_N_4_ materials before and after wear; (**A**) *R*_a_; (**B**) *R*_t_; and (**C**) *R*_sk_. In (**D**), compared increments in *R*_a_ and *R*_t_ after wear. The asterisks and *n.s.* represent *p* < 0.05 and *p* > 0.05, respectively.

The topographical analyses of the ceramic heads showed a general worsening of roughness parameters in comparison with the values measured on as-received surfaces (Figure 4). *R*_a_ significantly increased in both femoral head groups (*p* = 0.0087 and 0.0022 in ZTA and Si_3_N_4_) (Figure 4A). A clear increase in *R*_t_ was found in Si_3_N_4_ (*p* = 0.0043), whereas a similar change was not apparent for ZTA (*p* = 0.3874) (Figure 4B). The surfaces of both head materials became less negatively skewed after wear testing, indicating the generation of diminishing peaks, but no significant differences in *R*_sk_ were observed after the 5 Mc test (*p* = 0.2381 and 0.3312 for ZTA and Si_3_N_4_, respectively) (Figure 4C). Although mean values of wear-induced increments in *R*_a_ and *R_t_* were higher in Si_3_N_4_ than ZTA, the statistical comparisons between the two head groups showed no significance (*p* = 0.1602 and 0.1320) (Figure 4D).

**Figure 5:**
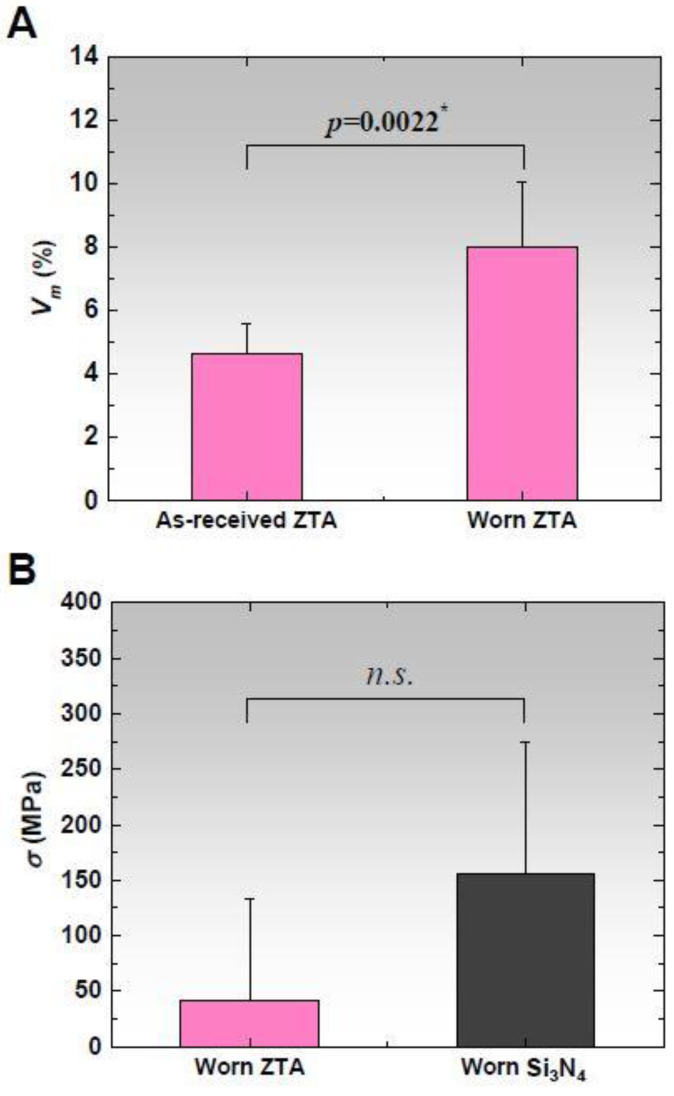
(**A**) Comparison of surface percentages of tetragonal-to-monoclinic phase transformation (*V_m_*) between the as-received (unworn) and worn ZTA heads; and (**B**) comparison of surface residual stress (*σ*) after wear between ZTA and Si_3_N_4_. The asterisks and *n.s.* represent *p* < 0.05 and *p* > 0.05, respectively.

Figure 5A compares the tetragonal-to-monoclinic phase transformation percentages (*V*_m_) between the as-received and worn ZTA head surfaces. The worn surfaces had significantly higher *V_m_* than the as-received heads (8.0% ± 2.1% vs. 4.7% ± 0.9%, *p* = 0.0022). Tensile residual stresses accumulated during testing in the Al_2_O_3_ matrix phase of ZTA (42.0 ± 91.0 MPa) and Si_3_N_4_ (155.5 ± 118.6 MPa) head surfaces, as shown in Figure 5B. Although the stress increase was higher in Si_3_N_4_ than in ZTA, no statistical differences were found between the head groups (*p* = 0.0931).

## 4. Discussion

This study presents the first comparison of the wear behavior and mass loss trends of Vit-E XLPE liners against ZTA and Si_3_N_4_ heads. Raman spectroscopy revealed significant losses in crystallinity on the articulation surfaces of the worn liners (Figure 3), which possibly occurred as a consequence of cumulative stresses during tribological loading (e.g., compression and shear). According to a previous study using the same brand of acetabular liners, an application of compressive strain demonstrated an increasing trend in surface crystallinity [25,28]. Therefore, the observed reduction in crystallinity after the test can be predominantly related to frictional shear stress leading to chain scission and destruction of the crystalline structure at the polymer surfaces. Nevertheless, the mean losses of weight and crystallinity were quite small (<3 mg and <3%, respectively), which were achieved due to the presence of intermolecular crosslinking and the antioxidant vitamin E. Oral et al. [29] reported that the in vitro mean wear rate of the same brand liners with an inner diameter of Ø28 mm was 0.78 ± 0.28 mg/Mc after a 5 Mc simulation. Their results were slightly higher than the present study (0.53 ± 0.24 and 0.49 ± 0.23 mg/Mc). This is likely because they chose a worst-case geometry—thinner liners (4.9 mm) coupled with cobalt–chrome heads.

Raman spectroscopy detected a substantial increase in the volume percentage of the monoclinic phase in ZTA (Figure 5A), but the transformed percentage during wear simulation was only 3.3%. It was previously demonstrated that this magnitude of phase transformation does not affect surface roughening in ZTA [30]. Note that the mean increments in *R*_a_ and *R*_t_ were fewer in ZTA than in Si_3_N_4_. (Figure 4D). Although there were no statistical significances in *R*_a_ and *R*_t_, ZTA seemed to retain a more favorable topography than Si_3_N_4_ (Figure 4D). Moreover, ZTA showed a lower magnitude of surface tensile stress after wear testing (Figure 5B) despite possessing lower toughness than Si_3_N_4_ (5.7 MPa m^1/2^ vs. 8–11 MPa m^1/2^) [12]. The higher Vickers hardness in ZTA achieved by the addition of Cr_2_O_3_ into the Al_2_O_3_ phase may account for the lower surface roughness compared to Si_3_N_4_ (19.1 GPa vs. 13–16 GPa [12]). Nevertheless, it is noteworthy that the smoother ZTA exhibited a higher mean wear rate of the Vit-E XLPE liners than Si_3_N_4_. Bowsher and Clarke [31] previously demonstrated that wear damage decreased with lower thermal conductivity femoral heads. This is because frictional heat generated at the bearing interface leads to thermal degradation of the serum lubricant, resulting in the precipitation of proteins. The presence of these proteins protects the articulation surface from abrasive wear [32,33]. This type of thermal artifact is more likely to occur in ZTA with its lower thermal conductivity than in Si_3_N_4_ (17 Wm^−1^K^−1^ vs. 30–40 Wm^−1^K^−1^ [12]). In other words, as compared to ZTA, Si_3_N_4_ with its higher thermal conductivity may present a somewhat more severe wear condition due to fewer protein precipitates. However, it remarkably exhibited less wear in the 5 Mc simulation test than ZTA (Figure 2). According to the study by Zhou et al. [34], the steady-state frictional coefficient of Si_3_N_4_ against itself in water was much smaller than that of Al_2_O_3_ against Al_2_O_3_ in water (0.001 vs. 0.08). Xu and Kato [35] reported that the wear mode of Si_3_N_4_ sliding in water changes from mechanically dominated to tribochemically dominated wear as sliding distance increases. They showed that a silica tribochemical layer is formed on the friction surface, which can significantly reduce friction during wear testing. The following chemical reactions are recognized as being present in friction testing of Si_3_N_4_ [35]:

Si_3_N_4_ +6H_2_O→3SiO_2_ + 4NH_3_ (1)

SiO_2_ + H_2_O→Si(OH)_4_. (2)

It is entirely likely that the formation of a “soft” silica layer in Si_3_N_4_ reduced friction, leading to the observed lower wear of Vit-E XLPE liners. However, such a superficial layer in Si_3_N_4_ may be more susceptible to microdamage (i.e., surface roughening) as compared to the harder surface of ZTA.

This study had a number of limitations. Femoral heads of a relatively small diameter (Ø28 mm) were used. These small heads were advantageous for Vit-E XLPE wear. Furthermore, the variability observed in the data may be the result of many machine variables. Simulated wear testing remains controversial, and factors such as simulator kinematics and their complexity, load application, utilization of a stationary head and moving liner or vice-versa, head on top or liner on top, multiple versus single station controls, lubricant composition, temperature, and circulation methods induce significant variability, which makes comparisons to other studies problematic. Moreover, this study suggests that current hip simulation standards inadequately represent the varied kinematics of the human gait. The common daily activities of standing, bending, walking, or running, coupled with rest periods of sitting or reclining, subject bearing couples to both a broad range of loads and repetitive stop-dwell-start cycles. In different studies on knee articulations, Affatato and co-workers [36,37] pointed out the limitations of the accepted hip simulator testing standards (i.e., ISO 14243-1,3). Such standards currently do not account for common clinically relevant conditions such as microseparation, malpositioning, edge loading, starved lubrication, and stop-motion, rest, and resume-motion sequences [38,39]. Each of these conditions has been shown to profoundly affect component wear and longevity.

## 5. Conclusions

The main outputs of this study can be summarized as follows:

(i) The wear data from hip simulation tests suggest that Si_3_N_4_ can be considered as a candidate material for femoral head components in innovative Ceramic-on-Polyethylene (CoP) hip joint articulations.
(ii) The wear performance of Si_3_N_4_ femoral heads was at least equivalent to that of the gold standard BIOLOX^®^*delta* after a 5 Mc test with negligible wear induced by both bioceramics against Vit-E XLPE counterparts.
(iii) Considering that the fracture toughness of Si_3_N_4_ components is superior to any other bioceramic so far tested [12], the present wear data represent positive news for the future development of long-lasting hip components.

## Figures and Tables

**Table 1:** Comparisons of cumulative weight loss of Vit-E XLPE acetabular cups coupled with ZTA (BIOLOX^®^*delta*) and Si_3_N_4_ (*MC2*^®^). *p*-values were evaluated by a non-parametric Mann–Whitney U test.

Test Cycles (Mc)	Mass Loss Average ± Standard Deviation (mg)		*p*-Value
	PE_ZTA	PE_Si_3_N_4_	
0.4	0.767 ± 0.2658	0.617 ± 0.2563	0.180
0.8	0.733 ± 0.6919	0.333 ± 0.2582	0.394
1.2	1.267 ± 0.9933	0.633 ± 0.2160	0.310
1.6	1.267 ± 0.9933	0.633 ± 0.2160	0.310
2.0	1.133 ± 0.9223	0.733 ± 0.2503	0.310
2.4	2.017 ± 1.0647	1.617 ± 0.6274	0.589
2.8	1.967 ± 1.1466	1.583 ± 1.1179	0.699
3.2	1.867 ± 1.1639	1.483 ± 0.8424	0.589
3.6	1.817 ± 1.0666	1.433 ± 1.0708	0.589
4.0	1.083 ± 0.9411	0.767 ± 0.1966	0.699
4.5	2.150 ± 1.1640	1.800 ± 1.0488	0.699
5.0	2.650 ± 1.2046	2.467 ± 1.1325	0.818

Statistical significance was set at *p* < 0.05
